# Are antibiotics substandard in Lebanon? Quantification of active pharmaceutical ingredients between brand and generics of selected antibiotics

**DOI:** 10.1186/s40360-020-0390-y

**Published:** 2020-02-22

**Authors:** Eva Hobeika, Joanna Farhat, Joseph Saab, Walid Hleihel, Samar Azzi-Achkouty, Georges Sili, Souheil Hallit, Pascale Salameh

**Affiliations:** 1 Faculty of Arts and Sciences, Holy Spirit University of Kaslik (USEK), Jounieh, Lebanon; 2 School of Engineering, Holy Spirit University of Kaslik (USEK), Jounieh, Lebanon; 3 Drug Information Center, Order of Pharmacists of Lebanon, Beirut, Lebanon; 4 Faculty of Medicine and Medical Sciences, Holy Spirit University of Kaslik (USEK), Jounieh, Lebanon; 5 INSPECT-LB: Institut National de Sante Publique, Epidemiologie Clinique et Toxicologie, Beirut, Lebanon; 6 Faculty of Pharmacy, Lebanese University, Beirut, Lebanon; 7 Faculty of Medicine, Lebanese University, Beirut, Lebanon

**Keywords:** Generic, Brand, Brand-generic substitution, Substandard, Ciprofloxacin hydrochloride, Amoxicillin trihydrate, Clavulanic acid, Antibiotic resistance, Therapeutic equivalence

## Abstract

**Background:**

In developing countries, brand-generic substitution is not based on validated scientific evidence that confirm the therapeutic equivalence of the generic to the originator. Rather, decisions are made based on the availability of generic medications. Substitution by inappropriate preparations applies to antibiotics, which may increase the risk of resistance in case of underdosing. This analytical study aims to dose and assess for the accuracy of labeling three oral antibiotic preparations, namely ciprofloxacin hydrochloride, amoxicillin trihydrate and amoxicillin trihydrate-clavulanate potassium, the active pharmaceutical ingredients (APIs) found in brand and generic tablets available on the Lebanese market.

**Methods:**

One brand and 4 generics of ciprofloxacin tablets, 3 generic amoxicillin tablets, and 1 brand and 4 generics of amoxicillin-clavulanic acid medications, were quantified, taking 2 batches of each. According to the United States Pharmacopeia (USP) guidelines, ultra-high pressure liquid chromatography was used to measure the APIs content within tablets. The USP required assay limit of the API was taken as the main comparison criteria.

**Results:**

Out of the 5 ciprofloxacin medications tested, all 5 were out of the 2% required range, thus being substandard. For amoxicillin, all 3 medications were within the 20% range. As for amoxicillin-clavulanic acid medications, 4 out of 5 medications met the 30% required range of clavulanic acid and one exceeded the claimed amount of clavulanic acid, while all 5 met the assay limit for amoxicillin.

**Conclusion:**

These findings raise safety and efficacy concerns, providing solid grounds for potential correlations of antibiotic resistance/substandard antibiotics.

## Background

The high cost of medications is one of the main barriers towards medicine access [1]. The unstable financial and economic situation in developing countries, along with increased costs of healthcare services have pushed for the selling of generic medications as alternatives to the 2.6 times more expensive brand medications (in the private sector) [1, 2]. A study conducted in 2012 by Cameron et al. showed that the switch from brand medications to generics in the private sector could result in an average of 60% cost savings [3].

To decrease pharmaceutical expenditures and guarantee a safe substitution, many developed countries introduced policies for generic drug substitution [2, 4]. In the United States of America, the Food & Drug Administration (FDA) defined the characteristics the generic drug must have compared to the brand: 1) contains the same active ingredient of the brand drug, 2) be similar in terms of strength, pharmaceutical form and way of administration, 3) has the same indications, 4) fulfills the same bioequivalence conditions, 5) fulfills the requirement of the batch in terms of identity, purity and quality, 6) is being manufactured in accordance with the standards of Good Manufacturing Practice, 7) is being compared to the brand in accordance with the standards of Good Laboratory Practices. When the generic drug fulfills the above-mentioned conditions, it can be considered as therapeutically equivalent to the brand, i.e. has the same efficacy and safety.

The WHO (World Health Organization) defines generic drugs as “pharmaceutical product, intended to be interchangeable with an innovator product manufactured without a license from the innovator company and marketed after the expiry date of the patent or other exclusive rights” [5]. Whereas brand medications are trade names that can only be manufactured and distributed by the company owning the patent, both of these drug categories are subject to quality assurance standards set by regulatory authorities. The United States FDA defines in its Orange Book pharmaceutical equivalents as “drug products in identical dosage forms and route of administration that contain identical amounts of the same active drug ingredient” [6]. Consequently, for generic and brand medications to be interchangeable, they must contain identical amounts of active pharmaceutical ingredients of similar quality.

At the international level, the International Council on Harmonization (ICH) sets clearly defined guidelines intended for the registration protocol of pharmaceutical products, in particular the third module entitled “quality” that details the quality assurance process [7]. In case generic or brand medications are inappropriately manufactured, they are termed substandard, substandard medications being defined by the WHO as “genuine medicines produced by manufacturer, not meeting quality specifications set to them by national standards”. It is important to note that this term is inclusive of both branded and generic medications [8]. In oral dosage forms, quantifying active pharmaceutical ingredient (API) assay is achieved through the analytical instrumentation of High Pressure Liquid Chromatography (HPLC). The potency of tablets – subsequently safety – is evaluated, and the tablets are considered substandard if the measures prove to be outside the accepted range of quality assurance [9].

Substandard medications can potentially affect clinical response for all diseases, and have been implicated in antibiotic resistance worldwide, raising alarming concerns among healthcare professionals. It is a type of drug resistance, where survival of usually sensitive bacteria, is observed after exposure to the antibiotic. This change in definitive survival is accelerated on the basis of factors related to human practices, with subsequent resistance selectivity in animals, humans and the environment [10]. Many theories have been put forward to explain the reason for this substantial increase in multiple pathogenic bacterial strains and their inevitable resistance to antibiotics worldwide, such as antibiotic misuse, antibiotic resistance and related treatment failures, in addition to poor quality of available medicines in developing countries (substandard drugs) with inadequate compliance with manufacturing practices, and lack of proper quality control [11]. Among the various classes of antibiotics, fluoroquinolones are worthy of attention as they contribute to the rapid emergence of resistance [12], along with another category that is the penicillin class, falling under the broad-spectrum antibiotics category, with its extensive use, low-cost and popularity in developing countries such as Lebanon [13].

In Lebanon, the general population has access to medicines through the private sector, and outpatients buy their medication mainly from community pharmacies. In August 2015, the Lebanese Ministry of Public Health (MOPH) issued ministerial decision no. One thousand, two hundred ninety-five that implemented the use of the unified medical prescription form among physicians, pharmacists and patients. This unified prescription gave the community pharmacist the right to substitute the brand medication by a lower-priced generic, unless otherwise specified by the physician. On the other hand, the Technical Committee within the MOPH is the authority that licenses medicines in Lebanon, and generics are considered as such, based on the dosage, dosage form, and active ingredient(s). The MOPH guidelines for the drug technical file submission are based on ICH’s module 3. The ministry’s online website has a section termed “Quality Assurance of Pharmaceutical Products”, under which fall these guidelines. Part 3.2.S.4 labeled “control of drug substance” is divided into the following guidelines: specification, analytical procedures, validation of analytical procedures, batch analyses and justification of specification [14]. However, in the absence of a central laboratory for assessing medications content, generics are registered in Lebanon based on files composed by the manufacturer and subsequent brand-generic substitution is performed based on the availability of medicines rather than on scientific evidence that accurately report that generic and brand are therapeutically equivalent. The MOPH defines pharmaceutical “equivalent” as referring to: “drug products, which contain the same active ingredient in the same strength (concentration) and dosage form, and is intended for the same route of administration.” The definition carries on by specifying conditions as: “In general, it has the same labeling and meets compendial and other standards of strength, quality, purity and identity.”

The aim of this study is the analytical control of 3 different antibiotic active pharmaceutical ingredients, allowing the quantitative assessment of their dosage as compared to the dosage stated on the label, hence being able to pinpoint substandard medications. This analytical evaluation of generic medications would set the pace to a broader objective, by taking a step further towards establishing a Lebanese equivalent to the US Approved Drug Products with Therapeutic Equivalence Evaluations – commonly known as the Orange Book. Therefore, this step would provide safer and more efficacious substitution of medications for patients seeking less expensive alternatives.

### What is already known about this subject?

In the United States, several studies were conducted comparing generic to brand medications and labeled dosage claim to actual experimental dosage, to evaluate therapeutic equivalence and potency of the medication under study. On a regional level, in the Middle East, a single study in Yemen evaluated the accuracy of dosage labeling of brand and generic antibiotic tablets, hence the need for the present study since there is no scientific evidence proving the therapeutic equivalence between brand and generic medications in Lebanon, particularly in the case of antibiotics and the increased risk of resistance.

### What this study adds?

The results of this study provided evidence that all tested brand and generic ciprofloxacin tablets, and one amoxicillin-clavulanic acid generic tablet, available on the Lebanese market, had a dosage deviation from what is mentioned on the label, exceeding the required range set by the United States Pharmacopeia (USP), and raising concerns of safety, effectiveness and therapeutic equivalence. Moreover, this study sheds the light on a new plausible cause underlying the increased occurrence of antibiotic resistance, through potential correlations between substandard medications and antibiotic resistance. Finally, it could pave the way for implementing a Lebanese equivalent to the US Orange Book.

## Methods

### Material

The following part details the materials used in the experimental and quantitative analysis of the ciprofloxacin, amoxicillin and amoxicillin/clavulanic acid APIs from the antibiotic tablets.

#### Chemical compounds

Table 1 enumerates the chemical compounds used throughout this study.

**Table 1:** Chemical compounds used during this study

Chemical Compounds	Structure^a^	Formula	CAS number	Purity	Supplier
Ciprofloxacin hydrochloride (reference standard)		C_17_H_19_ClFN_3_O_3_	85,721–33-1	Pharmaceutical secondary standard; certified reference material	Sigma-Aldrich
Methanol		CH_4_O	67–56-1	≥99.9% (Chromasolv, for HPLC)	Honeywell (Riedel-de Haën)
Acetonitrile		C_2_H_3_N	75–05-8	≥99.9% (Chromasolv gradient, for HPLC, gradient grade)	Honeywell (Riedel-de Haën)
Phosphoric acid		H_3_O_4_P	7664-38-2	98% (ACS reagent, reagent ISO)	Sigma-Aldrich
Triethylamine		C_6_H_15_N	121–44-8	99%	Scharlau
Amoxicillin trihydrate		C_16_H_19_N_3_O_5_S .3 H_2_O	61,336–70-7	Pharmaceutical Secondary Standard; Certified Reference Material	Sigma-Aldrich
Potassim clavulanate		C_8_H_8_NO_5_K	61,177–45-5	VETRANAL™ Analytical standard	Sigma-Aldrich

^a^Source for structures: American Chemical Society. Source for Ciprofloxacin structure: US FDA

Name and address of the companies that provided the compounds:

1) Name: Ibra HadadAddress: Jdeideh – Nahr El Mott, UniLeb Building, 2nd Floor.ibra@ibrahadad.comCompounds provided: ciprofloxacin hydrochloride (reference standard), acetonitrile, methanol, phosphoric acid, amoxicillin trihydrate, potassium clavulanate. Compounds purchased from: the United States of America

2) Name: BioDiagnosticAddress: El Bouchrieh Industrial City, Saint Jean Center, 2nd Floor.www.biodiagnostic-lb.comCompound provided: triethylamine.Compound purchased from: Spain

#### Instrumentation

The high pressure liquid chromatographic U-HPLC-DAD RS system model used (Thermo Ultimate 3000) is equipped with quaternary pump, auto sampler, and thermo stated column compartment with a variable wavelength detector controlled by the Chem station software. The HPLC system has a photodiode array detector (DAD) used for quantification studies. A C18 Thermo Scientific™ Hypersil GOLD™ HPLC column, small and meant for high pressure (100 × 2.1 mm), packed with particles of silica, spherical fully porous ultrapure (1.9 μm) was used as a stationary phase. For amoxicillin trihydrate and amoxicillin trihydrate-clavulanate potassium, the analysis was carried out on binary system using the C18 column (250 × 4.0 mm, 4 μm).

#### Standard and solvents

The standard used was Ciprofloxacin, pharmaceutical secondary standard; certified reference material purchased from the supplier Sigma-Aldrich, with CAS number: 855721–33-1. Amoxicillin and clavulanate potassium buffer standards were also provided from Sigma-Aldrich.

The solvents used were methanol, acetonitrile, sodium dihydrogen phosphate, triethylamine and phosphoric acid, all being of analytical grade. Ultra-pure water was prepared on a milli-Q purification system from Millipore (18 MΩ.cm). The uncertainty of the weighing balance was ±1 mg.

#### Mobile phase

As mobile phase, 87% of phosphoric acid buffer was added to 13% acetonitrile (ratio 87:13) in a graduated cylinder. The buffer was prepared as follows: 0.025 M phosphoric acid (equivalent to 2.85 mL of phosphoric acid in 2 L of ultrapure water), adjusted to a pH 3.0 using triethylamine solution.

For amoxicillin and amoxicillin trihydrate-clavulanate analysis, a degassed mixture of 5 volumes of methanol and 95 volumes of sodium dihydrogen phosphate (7.8 g L^− 1^) was used, adjusted to pH 4.5 using dilute ortho-phosphoric acid solution.

### Methods

The following part details the methods followed in the experimental and analytical protocol of the ciprofloxacin API from the 1 brand and 4 generic antibiotics (tablets), as well as the API from amoxicillin and amoxicillin-clavulanic acid medications.

#### Analysis

Analysis of ciprofloxacin was carried out on binary system using the high pressure C18 column (100 × 2.1 mm, 1.9 μm) with UV-detection of 278 nm. The column’s temperature was set at 30 °C using 1 μL injection volume. A C18 HPLC column (250 × 4.6 mm) packed with particles of silica gel, the surface of which has been modified with chemically-bonded octadecylsilyl groups (5 μm) was used as a stationary phase for amoxicillin and clavulanic acid analysis. The UV-detection is of 220 nm, with ambient temperature column and using 60 μL injection volume. The analysis was conducted using isocratic conditions; with the mobile phase mixture being consistent over the entire run time.

#### Calibration standards

Calibration standards have spanned the range from 5 to 200% of the expected API concentration in the experimental samples and 6 standards have been used to construct the calibration curve.

#### Normal unknown sample

The “normal” unknown sample was prepared in the 95–105% range for the analysis all APIs**.**

#### Standard stock solution

Standard stock solution was prepared by dissolving Ciprofloxacin hydrochloride standard equivalent to 40 mg of Ciprofloxacin in 100 mg of mobile phase (87:13 phosphoric acid buffer:acetonitrile). This standard solution was further diluted with mobile phase to get the standard solution concentration of Ciprofloxacin ranging from 1.0 mg.mL^− 1^ to 38 mg.mL^− 1^.

To each volumetric flask containing the diluted stock solution, 0.2 mL of 7% phosphoric acid was added. Finally, the 6 standard solutions and 1 normal unknown sample were emptied into small vials, to run through the U-HPLC. Regarding amoxicillin and clavulanic acid, the standard stock solution was prepared by dissolving amoxicillin trihydrate standard equivalent to 20 mg of clavulanic acid in 60 mL of ultra-pure water and then diluted to 100 mL with the same solvent. This stock solution was further diluted with water to get standard solution concentrations of amoxicillin ranging from 7.5 μg/mL to 150 μg/mL and those for clavulanic acid ranging from 1.5 μg/mL to 30 μg/mL.

#### Identity test

The retention times of the principal ciprofloxacin API peak in the chromatogram obtained with standard solutions.

### Unknown sample preparation

#### Equivalence of amount

The amount of ciprofloxacin, amoxicillin and clavulanic acid in solution for each sample of the generic and brand antibiotic should not be less than 85% of the amount declared on the label (*USP Monograph*). In this analysis, the weight of the unknown sample was taken as equivalent to the mass of one tablet, including approximately the total amount of APIs mentioned on the label.

#### Preparation of samples

Two batches of ciprofloxacin generic and brand antibiotic were picked. From each batch, 5 tablets were weighed and powdered. Therefore, 10 tablets in total were mixed and their average was accurately weighed using the diagonal sampling method: a systematic random picking technique (each 100 mg was taken from a side of the spread total powdered mass). This average mass, equal to the mass of 1 tablet, was transferred to a 500 mL volumetric flask. Mobile phase (87:13) was added until the meniscus trait, along with 0.1 mL of 7% phosphoric acid.

The flask was shaken and sonicated for 20 min to ensure proper homogenization. This was followed by a settlement time of 10 min. A small volume of the drug solution was then emptied into a beaker, and 15 mL were withdrawn using a sterile syringe, and filtered using a 0.45 μm filter tip.

From this filtered solution, 5 mL were withdrawn using a pipette and emptied into a 25 mL volumetric flask. Mobile phase solution (87:13) was added until the meniscus trait. The flask was put for about 10 s on the sonicator for homogenization. These steps were repeated for all 3 replicates. From this 25 mL volumetric flask, a small volume was withdrawn using a dropper and emptied to fill the U-HPLC vial.

The same process was applied for the sample preparation of amoxicillin and clavulanic acid, but with the following modifications: 10 tablets of each medicine was weighed and powdered, then a quantity of this powder, containing the equivalent of about 0.875 g of amoxicillin and 0.125 g of clavulanic acid, accurately weighed, was transferred into a 1000 mL volumetric flask, and 600 mL of ultrapure water was added and the flask was sonicated and shaken for 10 min. The solution was made up to volume with ultrapure water and filtered.

#### Repetition of samples

The same process mentioned above was repeated 3 times for every 2 batches of the same generic or brand antibiotic. Therefore, for each drug, 3 pills were typically analyzed. The same protocol was repeated for the 2 brand and 11 generic antibiotic drugs. All vials were put consecutively in the tray, and the U-HPLC instrument was run.

#### Peak detection and chromatograms

**Fig. 1:**
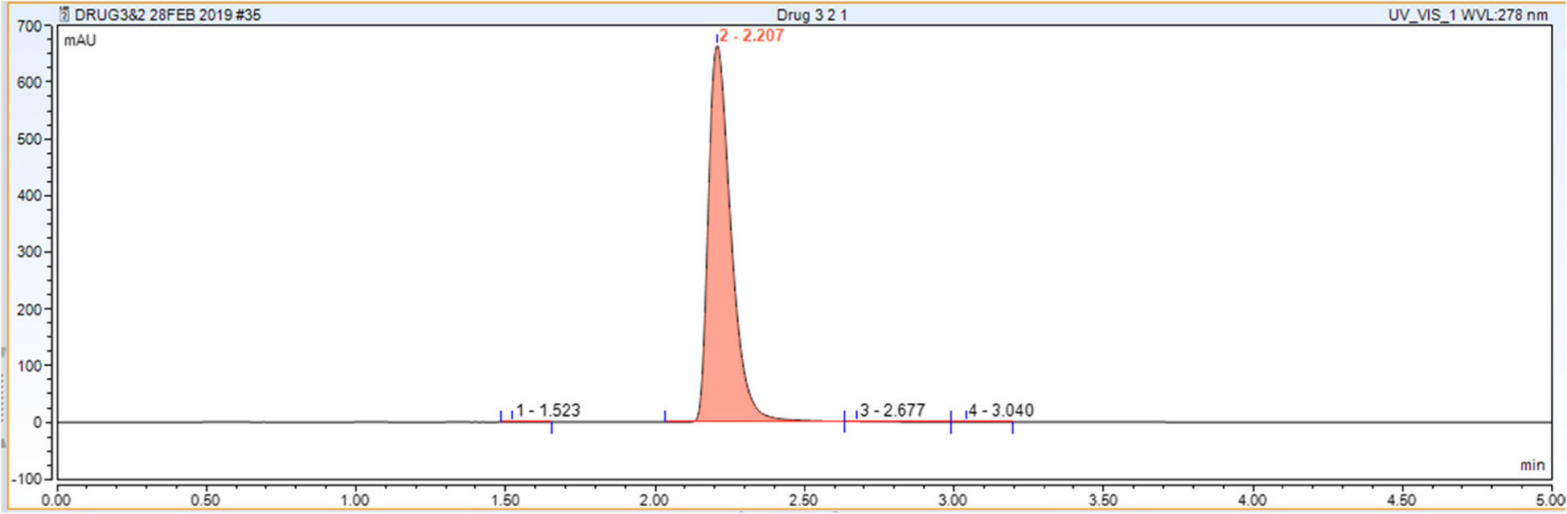
Chromatogram representing ciprofloxacin peak

The chromatogram peak corresponding to ciprofloxacin detection is in the range of 2–3.5 min. The reason why there is a shift in the retention time as compared to the Merck-Millipore ciprofloxacin assay of 10–12 min, is because of the use of the ultra-high pressure column. It is a ThermoFischer Scientific supplied Hypersil Gold C18 column, having a 100 × 2.1 mm length, with a 1.9 μm particle size, therefore subjecting the column to a much higher pressure, thus minimizing the time needed to detect the peak, and leading to a better optimization (Fig. 1).

**Fig. 2:**
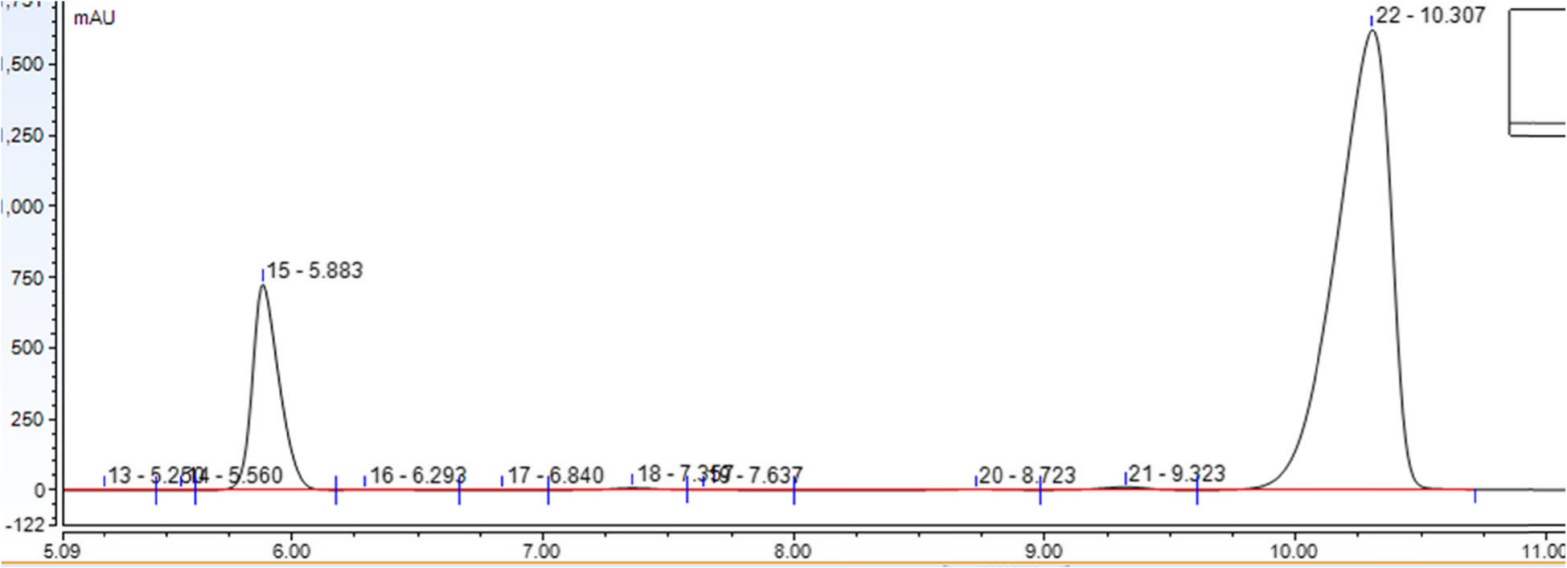
Chromatogram representing potassium clavulanate and amoxicillin trihydrate peaks

As for the second set of results, two chromatogram peaks were observed corresponding to the amoxicillin-clavulanic acid tablets. Although both APIs are concurrent, the detection of the peaks is characteristic of each API, with the potassium clavulanate peak retention time at 5.8 min, and the amoxicillin trihydrate peak retention time at 10.3 min (Fig. 2).

## Results

The results section will tackle the precision of the standard solutions measurements, the accuracy of measurements of ciprofloxacin normal unknown and finally the results of the ciprofloxacin assay. The following part details the results of the liquid chromatographic analysis of ciprofloxacin hydrochloride API molecule.

### Identity test and precision of measurements

This part will focus on the precision of the U-HPLC DAD RS and subsequently the results. It shows how close the measurements are to each other, by injecting the standard after every three unknown sample runs, as a quality check and assaying within 2% RSD of the 3 initial injections and be within the 0.3-min time range. The following measurements are targeted mainly to the precision of the instrument, showing that the results from the instrumentation are valid and deemed acceptable. Three injections of the external standard (and the unknown samples) have shown a peak area of APIs within the required 2.0% relative standard deviation (RSD), and the range of retention time is within 0.3 min (Additional file 1: Table S1, Additional file 2: Table S2 and Additional file 3: Table S3). Therefore, the following results obtained for the normal unknown samples and the unknown samples will be accepted since this preliminary control check is valid.

### Accuracy of measurements

This part focuses on the accuracy of the method applied and subsequently the accuracy of the results. A normal unknown solution is a solution that is prepared with a similar expected concentration of the unknown sample issued from the unknown sample preparation tablets. The relative deviation computes the ratio of difference between the experimental value obtained and the true expected value (Additional file 4: Tables S4, Additional file 5: Table S5, Additional file 6: Table S6 and Additional file 7: Table S7). It allows showing how much the experimental value has deviated from the true value. The results for the APIs show a good agreement between the true expected concentration and the experimental one with a relative deviation lower than the required range of deviation ±5%.

### Active pharmaceutical ingredient assay

The objective of this assay is the analytical control of the antibiotic API: ciprofloxacin (1 brand and 4 generic medications), amoxicillin (3 generic medications), and amoxicillin + clavulanic acid (1 brand and 4 generic medications), thus allowing the quantitative assessment of their dosage as compared to the dosage stated on the label. Included in the supplemental materials part are the calibration curves (Additional file 8: Figure S1, Additional file 9: Figure S2 and Additional file 10: Figure S3) corresponding to each run of sample sequence. From this curve and with the corresponding detection peak, the mass of API per tablet was calculated. The mean amount of API per tablet was then calculated based on the average of the 3 sample repetitions of each medication.

Tables 2, 3, 4, and 5 focus on the results of the API assay. The strategy of sampling used is the diagonal method, whereby the crushed antibiotic tablets or capsules were dispersed on a paper in a rectangular form and each 100 mg was taken in a diagonal mode (at opposite sides). The purpose behind this choice of sampling is to minimize the error coming from the sampling technique. Typically, an analysis of 3 tablets from each brand and generic antibiotic drug was performed (each weighed mass has an equivalent mass of 1 tablet).

**Table 2:** Amount in mg of ciprofloxacin in the tested weighed mass (runs 1, 2 and 3)

Medicine	Batch number	Mass of unknown sample (mg)	Mass of ciprofloxacin (CIP) per tablet (mg)	Mean amount of ciprofloxacin (CIP) per tablet (mg)	SD ^(a)^	RSD ^(b)^ (%)	Relative deviation© (%)	USP requirement
Drug 1.1 (run 1)	BXJ1KK2 BXJ22L1	762.7	464.8028	473.66	14.45	3.05	−5.27	98–102%
Drug 1.2		762.8	465.8469					
Drug 1.3		762.9	490.3392					
Drug 4.1	0802138 0802139	762.2	482.6215	474.38	7.25	1.53	−5.12	98–102%
Drug 4.2		762.4	468.9408					
Drug 4.3		762.4	471.5772					
Drug 5.1	M101 M102	763.5	461.0619	466.70	6.33	1.35	−6.66	98–102%
Drug 5.2		763.6	465.4737					
Drug 5.3		763.6	473.5532					
Drug 3.1 (run 2)	NOB51 N2KX1	824.2	537.1647	543.78	13.89	2.55	8.76	98–102%
Drug 3.2		824.4	534.4266					
Drug 3.3		824.2	559.7423					
Drug 3 (2).1	M12A1 M3R01	824.7	536.2957	525.33	11.15	2.12	5.07	98–102%
Drug 3 (2).2		824.8	514.0106					
Drug 3 (2).3		824.9	525.6963					
Drug 3 (3).1	N2 L01 N2KX1	822.8	524.1745	529.45	17.12	3.23	5.89	98–102%
Drug 3 (3).2		822.9	515.5925					
Drug 3 (3).3		822.9	548.5816					
Drug 2.1	4710 2966	773.9	485.4494	483.55	2.18	0.45	−3.29	98–102%
Drug 2.2		773.8	481.1726					
Drug 2.3		773.7	484.0210					
Drug2 [1].1(run 3)	5006 5006	774.2	493.3421	495.70	3.42	0.69	−0.86	98–102%
Drug 2 (1).2		774.1	499.6229					
Drug 2 (1).3		774.1	494.1410					
Drug 2 (2).1	4710 5006	772.9	491.9032	487.38	5.94	1.22	−2.52	98–102%
Drug 2 (2).2		772.8	480.6563					
Drug 2 (2).3		772.8	489.5944					

a: SD = $\sqrt{\frac{\sum \left(x-\overline{x}\right)}{n}}$ b: RSD = $\frac{SD\ }{\overline{X}}\times 100$ c: RD = $\frac{experimental- expected}{expected}$

**Table 3:** Amount in mg of amoxicillin in the tested weighed mass

Medicine	Mass of unknown sample	Mass of amoxicillinper pill	Mean amount of amox (mg)	SD ^(a)^	RSD ^(b)^ (%)	Relative deviation (%)©	USP requirement
Drug1.1	0.5886	544.29	547.14	2.60	0.47	9.42	90–120%
Drug1.2	0.581	549.42					
Drug1.3	0.5832	547.705					
Drug2.1	0.6058	554.11	556.90	4.55	0.81	11.38	90–120%
Drug2.2	0.6022	554.44					
Drug2.3	0.6068	562.16					
Drug3.1	0.6058	551.62	549.67	5.41	0.98	9.93	90–120%
Drug3.2	0.6022	553.83					
Drug3.3	0.6068	543.55					

a: SD = $\sqrt{\frac{\sum \left(x-\overline{x}\right)}{n}}$ b: RSD = $\frac{SD\ }{\overline{X}}\times 100$ c: RD = $\frac{experimental- expected}{expected}$

**Table 4:** Amount in mg of clavulanic acid in the tested weighed pill

Medicine	Mass of unknown sample	Mass of clavulanic acid (CA) per pill	Mean amount of clavulanic acid (CA) (in mg)	SD^(a)^	RSD ^(b)^ (%)	Relative deviation (%)©	USP requirement
Drug1.1	1478.1	150.452	147.44	2.91	1.97	17.95	90–120%
Drug1.2	1451.1	147.259					
Drug1.3	1471.8	144.623					
Drug2.1	1477.9	146.529	148.29	1.69	1.14	18.63	90–120%
Drug2.2	1544.8	148.447					
Drug2.3	1548.8	149.912					
Drug3.1	1463.0	141.884	144.37	2.56	1.77	15.5	90–120%
Drug3.2	1459.1	144.227					
Drug3.3	1474.1	147.013					
Drug4.1	1501.6	150.575	151.64	1.39	0.92	**21.31**	90–120%
Drug4.2	1501.3	151.137					
Drug4.3	1512.2	153.226					
Drug5.1	1513.3	147.668	147.73	0.88	0.6	18.18	90–120%
Drug5.2	1519	146.885					
Drug5.3	1532.6	148.665					

a: SD = $\sqrt{\frac{\sum \left(x-\overline{x}\right)}{n}}$ b: RSD = $\frac{SD\ }{\overline{X}}\times 100$ c: RD = $\frac{experimental- expected}{expected}$

**Table 5:** Amount in mg of amoxicillin in the tested weighed pill

Medicine	Mass of unknown sample	Mass of amoxicillin per pill	Mean amount of amoxicillin (in mg)	SD^(a)^	RSD^(b)^ (%)	Relative deviation© (%)	USP requirement
Drug1.1	1478.1	986.3631	969.46	15.39	1.58	10.79	90–120%
Drug1.2	1451.1	965.7939					
Drug1.3	1471.8	956.2252					
Drug2.1	1477.9	969.4269	983.60	12.97	1.31	12.41	90–120%
Drug2.2	1544.8	986.4853					
Drug2.3	1548.8	994.8977					
Drug3.1	1463.0	926.9293	952.01	28.73	3.01	8.80	90–120%
Drug3.2	1459.1	945.758					
Drug3.3	1474.1	983.3707					
Drug4.1	1501.6	1001.293	1003.43	6.30	0.62	14.67	90–120%
Drug4.2	1501.3	998.4876					
Drug4.3	1512.2	1010.529					
Drug5.1	1513.3	978.5518	985.95	6.49	0.65	12.68	90–120%
Drug5.2	1519	990.6797					
Drug5.3	1532.6	988.6453					

a: SD = $\sqrt{\frac{\sum \left(x-\overline{x}\right)}{n}}$ b: RSD = $\frac{SD\ }{\overline{X}}\times 100$ c: RD = $\frac{experimental- expected}{expected}$

For each of the weighed masses tested, a calculation of the total amount of ciprofloxacin in the medium using the declared amount of ciprofloxacin was computed. A relative deviation of ±2% was observed for all drugs (Table 2), thus being non-compliant with the declared contents of the unknown samples based on the USP requirement that clearly states that the sample fails analysis if the assay is under < 98% or > 102% of the stated API content.

In the case of amoxicillin, all 3 medications are within the 20% range (Table 3). USP requirement clearly states that sample fails analysis if the assay is under < 90% or > 120% of the stated API content.

In the case of amoxicillin-clavulanic acid medications (Tables 4 and 5), 4 out of 5 medications met the clavulanic acid’s 20% range, with one medication (drug 4) exceeding the declared content amount by 1.31% (21.31%) (Table 4).

## Discussion

This study offers insight into the quality of some of the antimicrobial generic drugs available in Lebanon. The results show a marked difference between the amount of API found in the generic drug tablets, and the actual dosage mentioned on the label. The drugs that have shown an amount less than that mentioned on the label can be contributors to antibiotic resistance since the dose taken by the patient is relatively less than that thought to be prescribed by the physician. Thus, not all bacteria are killed, leading to a consequential resistance of these remainder bacteria [12].

For amoxicillin, deviation from the labeled claim for potency was not found in the case of analysis of 3 different generic preparations. All 3 generic antibiotics were within the acceptable potency range of 90–120% set by the USP. Thus, a positive relative deviation, lower than 20%, is confirmed for all 3 medications. Therefore, these generic medications qualify as potent and safe, meeting the standard requirements.

For amoxicillin/clavulanate preparations, there is a deviation from the labeled claim for potency. Although a relative deviation, lower than 20%, is confirmed for the amoxicillin value in all 5 medications, the relative deviation lower than 20% is confirmed for 4 out of the 5 medications with regards to the clavulanic acid content. Drug 4 exceeded this range. In fact, based on healthcare professionals’ observation, patients who have taken drug 4 have reported a high rate of diarrhea. While patients taking other preparations with the same APIs did not report as much diarrhea; it is believed that the increased amount of clavulanic acid could be responsible for this undesired effect. A study by Evans et al. validates this assumption, since it mentions that antibiotic-associated diarrhea has a significantly higher occurrence rate (10–25%) when the treatment is amoxicillin-clavulanic acid, as compared to amoxicillin alone. Increased doses of amoxicillin-clavulanic acid can cause increased small intestine motility (through harmful effect of the natural microbiota), therefore leading to diarrhea [15].

Furthermore, deviation from the labeled claim for potency was found during analysis of 1 brand and 4 different generic drugs containing ciprofloxacin as API. USP requirement regarding ciprofloxacin hydrochloride states that the sample fails if the assay value is not within the 98–102% range of ciprofloxacin hydrochloride. A relative deviation exceeding the ±2% required range is observed for all four generics and even the brand antibiotic. Therefore, they are considered substandard preparations, and thus inappropriate for patient access.

An important note is that recent studies show that there is an increase of ESBL-producing bacteria. Specifically, fluoroquinolones are among the antibiotics that contribute to the rapid emergence of resistance. Adding to it the fact that certain generic antibiotics contain less than the required dose, resistance emergence would be favored even more. Undesired secondary effects are not reported at the time, but the main concern arising from these results is antibiotic resistance in case of all of these drugs since they contain doses less or more than the expected amount as per label claim. Thus, based on experimental numerical data, 6 out of the 13 tested brand and generics antibiotic medications cannot be substituted in place of their counterparts. These findings would lead to question the quality of the manufacturing process, affecting safety and therapeutic equivalence.

Moreover, the interchange cannot be done since the generic/brand drug does not fulfill the required guidelines set by the ICH and adopted by the MOPH for control of drug substances. More precisely, section 3.2.S.4.2 handling analytical procedures clearly states that “quantitative tests of active moiety in samples of drug substance”, and section 3.2.S.4.4 concerning batch analyses requires that a “description of batches and results of batch analyses should be provided” [16]. Therefore, these 6 drugs qualify as substandard and do not meet quality assurance requirements. No similar studies targeting ciprofloxacin analytical control have been conducted in Lebanon, but only one study was conducted in the Middle East, specifically in Yemen. It assessed 5 different brands of 500 mg ciprofloxacin hydrochloride tablets, bought from retail pharmacies, and results were however deemed satisfactory, with all brands meeting British Pharmacopeia and US Pharmacopeia requirements [17].

Therefore, our results highlight the added risk of having substandard antibiotics, in a community that considers antibiotics as a solution to almost all infectious cases, and tries to pay less from out of pocket. In a study conducted in Beirut, 42% out of the 319 participants reported buying antibiotics based on the pharmacist’s advice and not a physician’s prescription (18.8%). The cause behind this decision was believed that patients wanted to save time (55.7%) and money i.e. the cost of physician’s prescription (33.6%) [18]. The misuse of substandard antibiotics available on the market puts patients in a double trouble situation.

Consequently, to adequately address the safety of medication substitution, a proper guidance of healthcare professionals towards therapeutic equivalence of brand versus generic medication is required. The following strategic points aim to serve as guidelines to the making of clearer choices based on the safety of interchanging brand and generic medications. Recently, the Lebanese Order of Pharmacists (OPL), which is the official professional association of pharmacists in Lebanon and the legal partner of the MOPH, suggested to the MOPH a proposal regarding the safety of medication substitution. Its aim was to guide healthcare professionals towards the therapeutic equivalence of generic vs. brand medications.

The proposal included the following strategic points, to be discussed and agreed upon among all stakeholders:

- Unified patient information leaflet: The OPL suggested to the MOPH to use of a unified patient information leaflet, easily read and understood by the patient, on all medications that have a common indication. A template of the leaflet was also suggested.
- Reference drugs list: since the generics should be bioequivalent to the brand, the OPL proposed the creation of a reference drugs list. The reference drug is the drug to which all drugs are compared and that is approved by the MOPH upon the registration of a new drug; this aims at identifying discrepancies between multiple bioequivalent generics when compared to a single standard drug
- Laboratories following the Good Laboratory Practices (GLP): the MOPH should have a list of laboratories that follow the GLP and ensure that they continue to follow these practices and apply the terms of the biological match.
- List of interchangeable medications: the MOPH should have a list of all generic medications that are interchangeable with the brand, based on the therapeutic equivalence. All generics that are not listed can be prescribed by the doctor but cannot be considered by the pharmacist as a substitution option. This list constitutes basic scientific information for the community pharmacist for substituting medications in an effective and secure manner.
- Rating guidelines: the OPL specified a guideline for the substitution of generic medications by showing the results of the therapeutic equivalence between generics and inserting a rating that allows the possibility of drug substitution. As for narrow therapeutic index drugs and sustained-release medications, they are not to be replaced except under specific circumstances mentioned in the booklet according to scientific international standards.
- Practical pointers for generic substitution flowchart: Once all previous points are finalized, the OPL would develop a chart for the substitution of generic medications to guide community pharmacists and alert them upon substituting the abovementioned medications, taking into consideration the physiological characteristics of the patient (age, weight, liver function, kidney function and other critical conditions).

These strategic points would serve as guidelines for physicians and pharmacists in Lebanon, allowing them to make clearer choices and safely interchange brands and generics according to patients’ economic status. This would be the first step towards the establishment of an equivalent to the US “Orange Book”.

### Limitations of the study

This study focused mainly during the experimental part: on the therapeutic equivalence concept of the brand-generic substitution, without having tested for bioequivalence that is another main factor to be taken into consideration for substitution evidence. The assay for quantification of the medications focused on the analytical extraction and quantification of the API using U-HPLC. Another test that can be of value is the dissolution test, which was not performed during this analytical study and can give insights into the therapeutic equivalence. This sample size only included 13 medications; a bigger sample size would definitely make the results and conclusions more reliable and statistically significant. The standard solution preparation and the unknown sample preparation protocols are not identically the same, and the filtration of the solution before the final dilution was only done in the case of the unknown sample preparation. After filtration of the solution containing the API, excipients and impurities, an amount of API may have been lost by adhering to the filter. Therefore, there might be an error on the recuperated amount of API. Finally, this study mainly focused on the quantification assay and drew conclusions regarding potential antibiotic resistance. Additional in vitro studies (susceptibility of bacteria to antibiotics, etc...) may add further evidence towards the role of substandard medications and their effects on the biological and public health levels.

## Conclusions

The results of this study showed that the tested generic antibiotics belonging to the fluoroquinolones class, containing ciprofloxacin as the active pharmaceutical ingredient, were found to be substandard to the labeled dosage claim. In addition, one generic antibiotic containing amoxicillin and clavulanic acid was found to be out of range for its clavulanic acid content. These findings raise questions regarding the quality of medications available in Lebanon and open the door towards a better testing of generic medications, and the establishing of a database accessible to healthcare professionals for an informed and secure choice of medications.

## Supplementary information

**Additional file 1: Table S1.** Precision of peak detection of the standard solutions (runs 1, 2 and 3).

**Additional file 2: Table S2.** Precision of peak detection of the standard solutions (amoxicillin 500 mg medications).

**Additional file 3: Table S3.** Precision of peak detection of the standard solutions (amoxicillin and clavulanic acid medications).

**Additional file 4: Table S4.** Accuracy of measurements of ciprofloxacin solution (runs 1, 2 and 3).

**Additional file 5: Table S5.** Accuracy of measurements of amoxicillin solution.

**Additional file 6: Table S6.** Accuracy of measurements of clavulanic acid solution.

**Additional file 7: Table S7.** Accuracy of measurements of amoxicillin solution.

**Additional file 8: Figure S1.** Calibration curve for ciprofloxacin run 1.

**Additional file 9: Figure S2.** Calibration curve for ciprofloxacin run 2.

**Additional file 10: Figure S3.** Calibration curve for ciprofloxacin run 3.

## Data Availability

All data generated or analysed during this study are included in this published article and its supplementary information files.

## Abbreviations

API: Active pharmaceutical ingredients
DAD: Photodiode array detector
ESBL: Extended Spectrum Beta Lactamase
FDA: Food & Drug Administration
HPLC: High Pressure Liquid Chromatography
ICH: International Council on Harmonization
MOPH: Lebanese Ministry of Public Health
OPL: Order of Pharmacists of Lebanon
RSD: Relative standard deviation
U-HPLC: Ultra High Performance Liquid Chromatography
USP: United States Pharmacopeia
WHO: World Health Organization
