# Supplementary material for: Are antibiotics substandard in Lebanon? Quantification of active pharmaceutical ingredients between brand and generics of selected antibiotics
Source: BMC Pharmacol Toxicol. 2020 Feb 22;21:15. doi: 10.1186/s40360-020-0390-y (PMC7036234; doi:10.1186/s40360-020-0390-y)
Supplement: Supplementary file 1 — Additional file 1: Table S1. Precision of peak detection of the standard solutions (runs 1, 2 and 3). [file 40360_2020_390_MOESM1_ESM.docx]

Supplementary table 1: Precision of peak detection of the standard solutions (runs 1, 2 and 3)

| Standard solutions | C_cip (mg.ml_^-1^_)_ | Mean Surface (CIP) | (a) SD Surface (CIP) | (b) RSD (%) Surface (CIP) |
| --- | --- | --- | --- | --- |
| (Run 1) Std1 | 0.01064 | 2.553525 | 0.012957 | 0.507414 |
| Std2 | 0.04986 | 12.35453 | 0.053933 | 0.436541 |
| Std3 | 0.09641 | 24.6079 | 0.165718 | 0.673436 |
| Std4 | 0.32154 | 83.56027 | 0.742253 | 0.888285 |
| Std5 | 0.35464 | 90.9215 | 0.577054 | 0.634673 |
| Std6 | 0.37544 | 97.10957 | 0.332863 | 0.342771 |
| (Run 2) Std1 | 0.01094 | 2.6728 | 0.043604 | 1.631403 |
| Std2 | 0.05097 | 12.533 | 0.131176 | 1.046644 |
| Std3 | 0.09712 | 23.91545 | 0.398102 | 1.664622 |
| Std4 | 0.32993 | 81.3178 | 0.202818 | 0.249414 |
| Std5 | 0.36117 | 91.14583 | 0.676516 | 0.742235 |
| Std6 | 0.38243 | 96.04493 | 0.443322 | 0.461578 |
| (Run 3) Std1 | 0.01094 | 2.696975 | 0.015834 | 0.587087 |
| Std2 | 0.05097 | 12.52257 | 0.110082 | 0.879072 |
| Std3 | 0.09712 | 23.7824 | 0.054253 | 0.228122 |
| Std4 | 0.32993 | 81.7845 | 1.468538 | 1.795619 |
| Std5 | 0.36117 | 91.49785 | 1.391409 | 1.520701 |
| Std6 | 0.38243 | 95.50233 | 0.395711 | 0.414347 |

a: SD = $\sqrt{\frac{\sum\left( \boldsymbol{x} - \overline{\boldsymbol{x}} \right)}{\boldsymbol{n}}}$ b: RSD = $\frac{\boldsymbol{SD}}{\overline{\boldsymbol{X}}} \times100$
