# Supplementary material for: Are antibiotics substandard in Lebanon? Quantification of active pharmaceutical ingredients between brand and generics of selected antibiotics
Source: BMC Pharmacol Toxicol. 2020 Feb 22;21:15. doi: 10.1186/s40360-020-0390-y (PMC7036234; doi:10.1186/s40360-020-0390-y)
Supplement: Supplementary file 2 — Additional file 2: Table S2. Precision of peak detection of the standard solutions (amoxicillin 500 mg medications). [file 40360_2020_390_MOESM2_ESM.docx]

Supplementary table 2: Precision of peak detection of the standard solutions (amoxicillin 500mg medications)

| Standard solutions | C_AMOX (mg ml_^-1^_)_ | Mean Surface AMOX | SDSurface (AMOX) ^(a)^ | RSD (%) Surface (AMOX) ^(b)^ |
| --- | --- | --- | --- | --- |
| Std1 | 0.0053300 | 2.3541 | 7.07E-05 | 0.003004 |
| Std2 | 0.0251934 | 11.24345 | 0.100338 | 0.892417 |
| Std3 | 0.0522912 | 22.9761 | 0.044548 | 0.193887 |
| Std4 | 0.1525897 | 69.10595 | 0.128481 | 0.185919 |
| Std5 | 0.1737888 | 78.1343 | 0.077499 | 0.099187 |
| Std6 | 0.2065166 | 93.6727 | 0.152452 | 0.16275 |

a: SD = $\sqrt{\frac{\sum\left( x - \overline{x} \right)}{n}}$ b: RSD = $\frac{SD}{\overline{X}} \times100$
