# Supplementary material for: Are antibiotics substandard in Lebanon? Quantification of active pharmaceutical ingredients between brand and generics of selected antibiotics
Source: BMC Pharmacol Toxicol. 2020 Feb 22;21:15. doi: 10.1186/s40360-020-0390-y (PMC7036234; doi:10.1186/s40360-020-0390-y)
Supplement: Supplementary file 3 — Additional file 3: Table S3. Precision of peak detection of the standard solutions (amoxicillin and clavulanic acid medications). [file 40360_2020_390_MOESM3_ESM.docx]

Supplementary table 3: Precision of peak detection of the standard solutions (amoxicillin and clavulanic acid medications)

| Standard solutions | C_AMOX (mg ml_^-1^_)_ | Mean Surface AMOX | SDSurface (AMOX) ^(a)^ | RSD (%) Surface (AMOX) ^(b)^ |  | C_CA (mg ml_^-1^_)_ | Mean Surface (CA) | SDSurface (CA) ^(a)^ | RSD (%) Surface (CA) ^(b)^ |
| --- | --- | --- | --- | --- | --- | --- | --- | --- | --- |
| Std1 | 0.0053300 | 2.3541 | 7.07E-05 | 0.003004 |  | 0.0014 | 2.1703 | 0.0035 | 0.054074 |
| Std2 | 0.0251934 | 11.24345 | 0.100338 | 0.892417 |  | 0.0083 | 12.9209 | 0.00786 | 0.021295 |
| Std3 | 0.0522912 | 22.9761 | 0.044548 | 0.193887 |  | 0.0153 | 23.9858 | 0.05638 | 0.082269 |
| Std4 | 0.1525897 | 69.10595 | 0.128481 | 0.185919 |  | 0.0427 | 66.7644 | 0.04425 | 0.0232 |
| Std5 | 0.1737888 | 78.1343 | 0.077499 | 0.099187 |  | 0.0500 | 78.0201 | 0.02831 | 0.012702 |
| Std6 | 0.2065166 | 93.6727 | 0.152452 | 0.16275 |  | 0.0575 | 89.7364 | 0.06867 | 0.026786 |

a: SD = $\sqrt{\frac{\sum\left( x - \overline{x} \right)}{n}}$ b: RSD = $\frac{SD}{\overline{X}} \times100$
