# Supplementary material for: Are antibiotics substandard in Lebanon? Quantification of active pharmaceutical ingredients between brand and generics of selected antibiotics
Source: BMC Pharmacol Toxicol. 2020 Feb 22;21:15. doi: 10.1186/s40360-020-0390-y (PMC7036234; doi:10.1186/s40360-020-0390-y)
Supplement: Supplementary file 4 — Additional file 4: Table S4. Accuracy of measurements of ciprofloxacin solution (runs 1, 2 and 3). [file 40360_2020_390_MOESM4_ESM.docx]

Supplementary table 4: Accuracy of measurements of ciprofloxacin solution (runs 1, 2 and 3)

|  | True Expected Concentration CIP (mg.ml-^1^) | Experimental Concentration CIP (mg.ml-^1^) | Relative deviation (%) ^(a)^ | USP Accuracy requirement (%) |
| --- | --- | --- | --- | --- |
| Normal Unknown (run 1) | 0.2010182 | 0.1960401 | 2.47% | ±5% |
| Normal Unknown  (run 2) | 0.2027911 | 0.1968677 | 2.92% | ±5% |
| Normal Unknown  (run 3) | 0.2027911 | 0.1980150 | 2.35% | ±5% |

a: RD = $\frac{experimental -expected}{expected}$
