# Supplementary material for: Are antibiotics substandard in Lebanon? Quantification of active pharmaceutical ingredients between brand and generics of selected antibiotics
Source: BMC Pharmacol Toxicol. 2020 Feb 22;21:15. doi: 10.1186/s40360-020-0390-y (PMC7036234; doi:10.1186/s40360-020-0390-y)
Supplement: Supplementary file 5 — Additional file 5: Table S5. Accuracy of measurements of amoxicillin solution. [file 40360_2020_390_MOESM5_ESM.docx]

Supplementary table 5: Accuracy of measurements of amoxicillin solution

|  | True Expected Concentration AMOX (mg ml-^1^) | Experimental Concentration AMOX (mg ml-^1^) | Relative deviation (%) ^(a)^ | USP Accuracy requirement (%) |
| --- | --- | --- | --- | --- |
| Normal Unknown | 0.1046 | 0.1058 | 0.8 | ±5% |

a: RD = $\frac{experimental -expected}{expected}$
