# Supplementary material for: Are antibiotics substandard in Lebanon? Quantification of active pharmaceutical ingredients between brand and generics of selected antibiotics
Source: BMC Pharmacol Toxicol. 2020 Feb 22;21:15. doi: 10.1186/s40360-020-0390-y (PMC7036234; doi:10.1186/s40360-020-0390-y)
Supplement: Supplementary file 6 — Additional file 6: Table S6. Accuracy of measurements of clavulanic acid solution. [file 40360_2020_390_MOESM6_ESM.docx]

Supplementary table 6: Accuracy of measurements of clavulanic acid solution

|  | True Expected Concentration CA (mg ml-^1^) | Experimental Concentration CA (mg ml-^1^) | Relative deviation (%) ^(a)^ | USP Accuracy requirement (%) |
| --- | --- | --- | --- | --- |
| Normal Unknown 1 | 0.0225 | 0.0226 | 0.45 | ±5% |
| Normal Unknown 2 | 0.0288 | 0.0290 | 0.60 | ±5% |

a: RD = $\frac{experimental -expected}{expected}$
