# Supplementary material for: Are antibiotics substandard in Lebanon? Quantification of active pharmaceutical ingredients between brand and generics of selected antibiotics
Source: BMC Pharmacol Toxicol. 2020 Feb 22;21:15. doi: 10.1186/s40360-020-0390-y (PMC7036234; doi:10.1186/s40360-020-0390-y)
Supplement: Supplementary file 7 — Additional file 7: Table S7. Accuracy of measurements of amoxicillin solution. [file 40360_2020_390_MOESM7_ESM.docx]

Supplementary table 7: Accuracy of measurements of amoxicillin solution

|  | True Expected Concentration AMOX (mg ml-^1^) | Experimental Concentration AMOX (mg ml-^1^) | Relative deviation (%) ^(a)^ | USP Accuracy requirement (%) |
| --- | --- | --- | --- | --- |
| Normal Unknown 1 | 0.1207 | 0.1211 | 0.33 | ±5% |
| Normal Unknown 2 | 0.1548 | 0.1551 | 0.19 | ±5% |

a: RD = $\frac{experimental -expected}{expected}$
