# Supplementary material for: Are antibiotics substandard in Lebanon? Quantification of active pharmaceutical ingredients between brand and generics of selected antibiotics
Source: BMC Pharmacol Toxicol. 2020 Feb 22;21:15. doi: 10.1186/s40360-020-0390-y (PMC7036234; doi:10.1186/s40360-020-0390-y)
Supplement: Supplementary file 9 — Additional file 9: Figure S2. Calibration curve for ciprofloxacin run 2. [file 40360_2020_390_MOESM9_ESM.docx]

Supplementary figure 2: Calibration curve for ciprofloxacin run 2.
